# Association between circulating immune cells and the risk of prostate cancer: a Mendelian randomization study

**DOI:** 10.3389/fendo.2024.1358416

**Published:** 2024-02-09

**Authors:** Xuexue Hao, Congzhe Ren, Hang Zhou, Muwei Li, Hao Zhang, Xiaoqiang Liu

**Affiliations:** Department of Urology, Tianjin Medical University General Hospital, Tianjin, China

**Keywords:** prostate cancer, immune cells, Mendelian randomization study, causality, reverse causality

## Abstract

**Background:**

There is still limited research on the association between immune cells and the risk of prostate cancer. Further investigations are warranted to comprehend the intricate associations at play.

**Methods:**

We used a bidirectional two-sample Mendelian randomization (MR) analysis to investigate the causal relationship between immune cell phenotypes and prostate cancer. The summary data for immune cell phenotypes was derived from a study cohort, including 3,757 individuals from Sardinia with data on 731 immune cell phenotypes. The summary data for prostate cancer were obtained from the UK Biobank database. Sensitivity analyses were conducted, and the combination of MR-Egger and MR-Presso was used to assess horizontal pleiotropy. Cochran’s Q test was employed to evaluate heterogeneity, and the results were subjected to FDR correction.

**Results:**

Our study identified two immune cell phenotypes significantly associated with the risk of prostate cancer, namely CD25 on naive-mature B cells (OR = 0.998, 95% CI, 0.997-0.999, *P* = 2.33E-05, FDR = 0.017) and HLA DR on CD14- CD16- cells (OR = 1.001, 95% CI, 1.000-1.002, *P* = 8.01E-05, FDR = 0.03). When adjusting FDR to 0.2, we additionally found six immune cell phenotypes influencing the incidence of prostate cancer. These include FSC-A on B cells (OR = 1.002, 95% CI, 1.001-1.002, *P* = 7.77E-04, FDR = 0.133), HLA DR on plasmacytoid dendritic cells (OR = 1.001, 95% CI, 1.000-1.001, *P* = 0.001, FDR = 0.133), CD14+ CD16- monocyte % monocytes (OR = 1.002, 95% CI, 1.001-1.003, *P* = 0.001, FDR = 0.133), and HVEM on effector memory CD4+ T cells (OR = 1.001, 95% CI, 1.000-1.002, *P* = 0.002, FDR = 0.169), which are positively correlated with the risk of prostate cancer. Conversely, CD25 on IgD+ B cells (OR = 0.998, 95% CI, 0.997-0.999, *P* = 0.002, FDR = 0.169) and Monocytic Myeloid-Derived Suppressor Cells AC (OR = 0.999, 95% CI, 0.999-1.000, *P* = 0.002, FDR = 0.17) are negatively correlated with the risk of prostate cancer.

**Conclusion:**

This study has revealed causal relationships between immune cell phenotypes and prostate cancer, supplying novel insights that might aid in identifying potential therapeutic targets of prostate cancer.

## Introduction

Prostate cancer is the second most common cancer in males, with an incidence rate second only to lung cancer. It is also the most common cancer in the male urinary system (1). The incidence of prostate cancer increases with the age of males (2). Additionally, there are significant differences in the incidence of prostate cancer based on race and geographic location. There is a 40-fold difference in incidence rates between African American males with the highest incidence and native Asian males with the lowest incidence in the United States (3). Western Europe, Northern Europe, North America, and other countries are high-incidence regions for prostate cancer, while regions such as Asia and North Africa have relatively lower incidence rates of prostate cancer (4). In addition to recognized age factors, risk factors for prostate cancer also include genetics, baldness, height, and others. Additionally, there are modifiable risk factors, including diet, smoking, and alcohol consumption (5).

An increasing number of studies have found a complex and close association between the immune system and cancer (6, 7). Many immunotherapies, such as immune checkpoint inhibitors or direct targeting of the tumor immune microenvironment, are used in cancer treatment, focusing on various immune cells within the immune system (8). Under normal circumstances, immune cells exert anti-tumor effects through immune surveillance and immune cytotoxicity. However, under certain conditions, certain immune cells may also promote the progression of tumors (9). This dual effect of immune cells occurs in various cancers. Studies have found a positive correlation between higher levels of FOXP3+ T cells mediating immune tolerance and lower levels of CD8+ T cells mediating cytotoxicity with the risk of breast cancer, colorectal cancer, and lung cancer in normal healthy populations (10). Similarly, research suggests that changes in the composition of immune cell tissues are linked to an elevated or reduced risk of specific cancers (11).. Immune cells also play a crucial role in prostate cancer (12–14). Studying the connection between immune cells and prostate cancer will contribute to exploring the mechanisms of prostate cancer, providing more potential treatment methods, and alleviating the burden on patients and society. Currently, there is still limited research on the association between immune cells and the risk of prostate cancer (15). More studies are needed to understand the complex connections involved.

Mendelian randomization (MR) is a method that utilizes genetic variations as instrumental variables (IVs) to assess observed causal relationships (16). The purpose of this approach is to simulate a randomized controlled trial, mitigating the influence of potential confounding factors in observational studies (17). Traditional observational studies determine disease risk factors by examining the relationship between exposure and outcomes. However, these studies may be limited in drawing valid causal conclusions due to confounding factors or reverse causation (18). Compared to traditional observational studies, MR is valuable for investigating causal relationships between risk factors and clinical diseases because genetic variations are randomly assigned at conception, typically unrelated to confounding factors, and unaffected by reverse causation (19). Our research aims to explore a causal relationship between immune cell traits and prostate cancer through a comprehensive two-sample bidirectional MR analysis.

## Methods

### Study design

**Figure 1:**
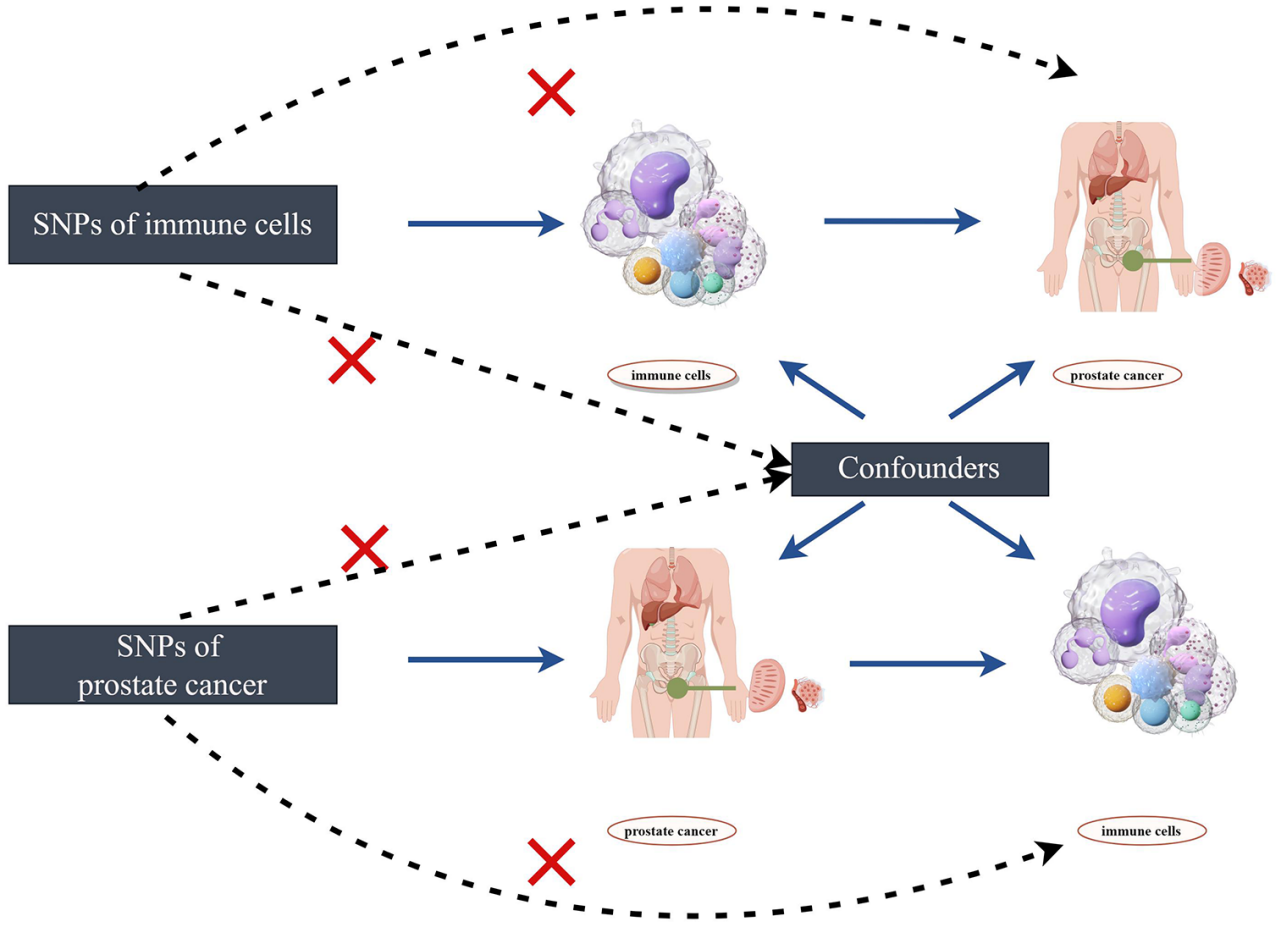
The design of bidirectional Mendelian randomization (MR) study by Figdraw.

The flowchart of the two-sample bidirectional Mendelian randomization (MR) analysis for immune cell phenotypes and the risk of prostate cancer is depicted in **Figure 1**. Initially, we employ immune cell phenotypes as the exposure to analyze which immune cell phenotypes may have potential causal relationships with the risk of prostate cancer. Subsequently, we use prostate cancer as the exposure and explore the potential reverse causal relationships with immune cell phenotypes. Single nucleotide polymorphisms (SNPs) are utilized as IVs in the study. The selected IVs satisfy three crucial assumptions: (1) IVs are associated with the risk exposure. (2) IVs are unrelated to any confounding factors influencing the exposure-outcome relationship. (3) IVs can only affect the outcome through the exposure and not through any other pathways (20). Any IVs violating the three major assumptions will be excluded.

### Data sources

#### Immunology-related GWAS data sources

Our research data is derived from open GWAS databases and the UK Biobank database. The studies involved have all been approved by the local ethics committee. This study did not collect new data and does not require new ethical approval.

The summary statistics of immune cell phenotypes are derived from the GWAS database. GWAS data identifier from GCST90001391 to GCST90002121 (https://www.ebi.ac.uk/gwas/studies/GCST90002121). A cohort study involving 3,757 Sardinian individuals reported data on 22 million variants for 731 immune cell phenotypes. The 731 immune cell phenotypes consist of 118 absolute cell counts (AC), 192 relative counts (RC), 389 median fluorescence intensities (MFIs) of surface antigens, and 32 morphological parameters (MP).

This study involved the collection of peripheral blood from blood donors, which was then subjected to flow cytometry analysis following antibody staining. This process allowed for the identification and quantification of different cell subpopulations (21).

#### GWAS data sources for prostate cancer

Prostate cancer data were obtained from the UK Biobank database, comprising a study population of 462,933 individuals of European descent. The case group consisted of 3,269 individuals, and the control group included 459,664 individuals, involving 9,851,867 SNPs.

#### Selection of IVs

We set the threshold for SNPs related to immune cell phenotypes at *P* < 1 × 10^-5^. Additionally, we conducted a Linkage Disequilibrium (LD) check on these SNPs (r^2 = ^0.001 and kb = 10,000). The values of r^2^ or kb represent the degree of linkage disequilibrium between two loci, indicating that if there is LD between two loci, their allele frequencies are not independent but correlated in some way. For SNPs related to prostate cancer, we applied a threshold of *P* < 5 × 10^-8^ and similarly performed LD checks (r^2 = ^0.001 and kb = 10,000). We calculated the F-statistic for each SNP, and SNPs with low F-values (< 10) were removed as IVs to assess IV strength and mitigate weak instrument bias (22). Finally, we utilized the PhenoScanner database to exclude SNPs associated with potential confounding variables.

#### Statistical analysis

In order to explore the causal relationship between immune cell phenotypes and the risk of prostate cancer, this study primarily utilized the TwoSampleMR and MRPRESSO packages in R (4.2.3). The main conventional MR analysis methods employed included Inverse Variance Weighting (IVW), MR-Egger, Weighted Median, Weighted Mode, and MR-Presso. Depending on the specific situation, choose random or fixed-effect IVW. IVW, as the primary analytical method, aims to estimate the causal relationship between exposure factors and outcomes by combining the effects of various genetic variations, providing a comprehensive causal estimation. However, IVW has limitations as it relies on the three fundamental assumptions mentioned earlier and can only avoid the influence of confounding factors in the absence of horizontal pleiotropy. Therefore, when using the IVW method, the potential for horizontal pleiotropy must be considered (23).

In this study, we employed a combination of MR-Egger and MR-Presso to assess the presence of horizontal pleiotropy (*P* < 0.05 considered to indicate horizontal pleiotropy). Additionally, Cochran’s Q test was utilized to assess heterogeneity among the selected SNPs *(P* < 0.05 considered to indicate heterogeneity). Compared to MR-Egger, which detects and quantifies the degree of horizontal pleiotropy through intercept testing, MR-Presso can identify and address outliers beyond horizontal pleiotropy (24).

To explore reverse causality, the same methods were used for reverse MR analysis of immune cell phenotypes and prostate cancer. Moreover, considering the issue of multiple testing, FDR correction was performed using the online tool Bioladder. According to previous studies, FDR < 0.2 is considered suggestive of a causal relationship, while FDR < 0.05 is considered to indicate a significant causal relationship.

## Results

### The causal effect of immunophenotypes on prostate cancer

**Figure 2:**
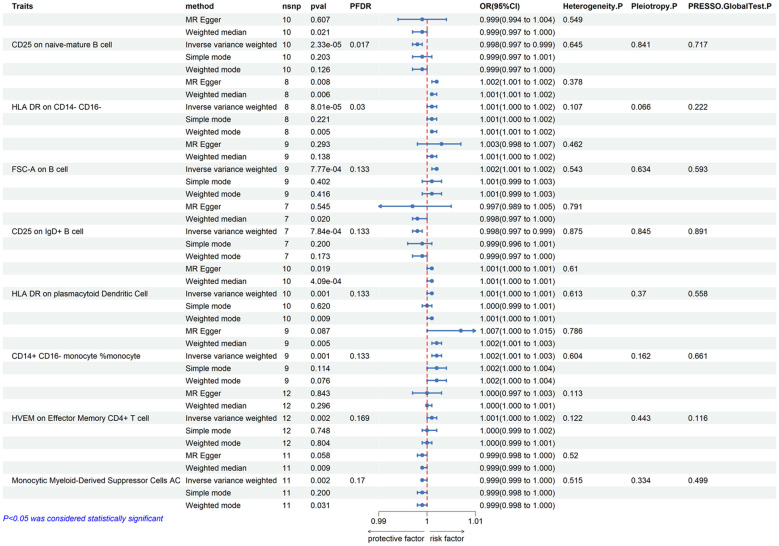
Forest plots showed the causal effect of immunophenotypes on prostate cancer. nsnp, nonsynonymous single-nucleotide polymorphism; OR, odds ratio; CI, confidence interval; PFDR, P value corrected by FDR.

**Figure 3:**
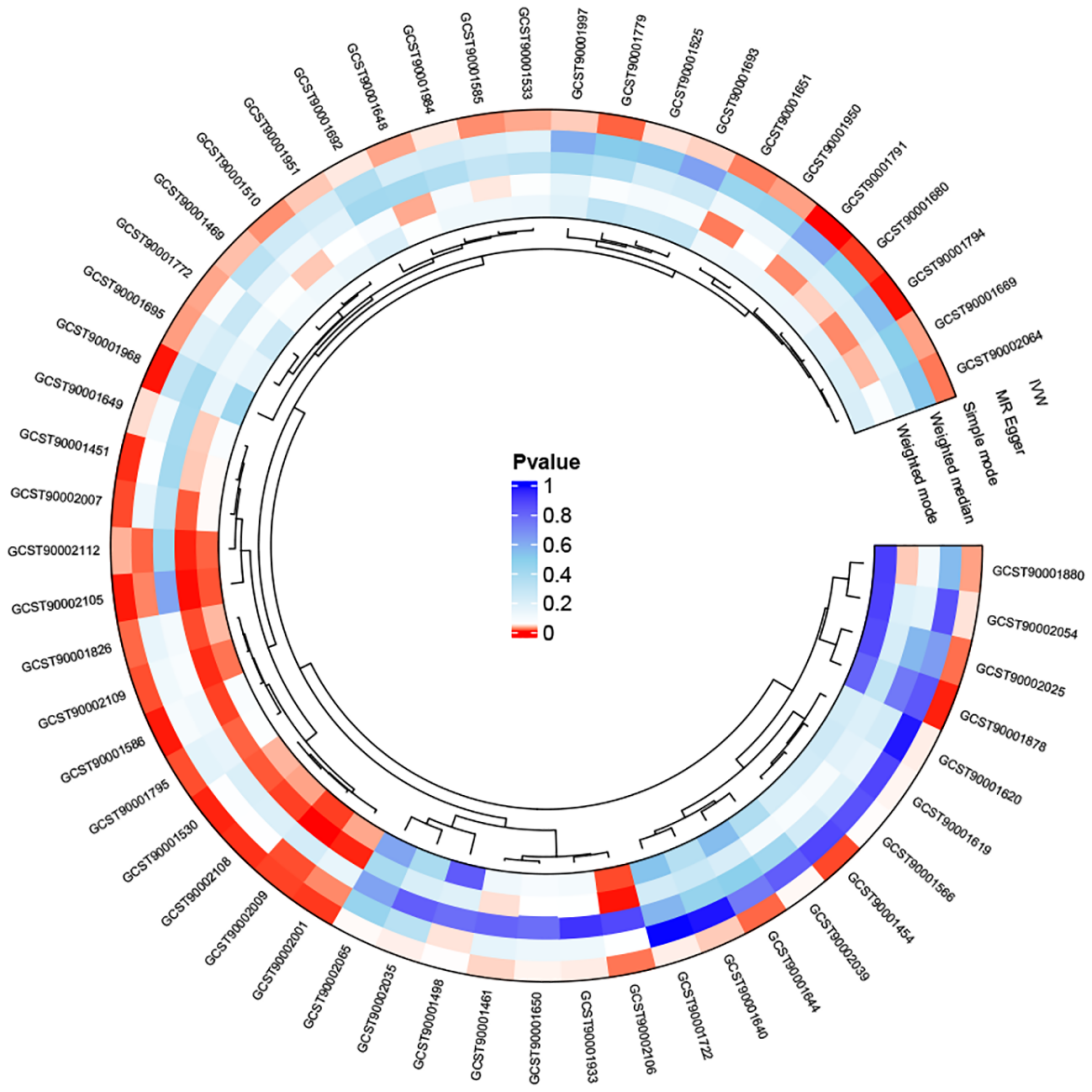
The heatmap depicting the IDs of immune cell phenotypes with positive results and the p-values from the sensitivity analysis: The outer circle represents the IDs of immune cell phenotypes, while the inner circle uses different colors to indicate the p-values of different sensitivity analysis results.

We first analyzed the causal effects of 731 immune cell phenotypes as exposure variables on prostate cancer, and the results of the analysis are shown in **Figure 2**. Our research findings revealed that two immune cell phenotypes are significantly associated with the risk of prostate cancer. Specifically, CD25 on naive-mature B cells (OR = 0.998, 95% CI, 0.997-0.999, *P* = 2.33E-05, FDR = 0.017) shows a significant negative correlation with the risk of prostate cancer, while HLA DR on CD14- CD16- cells (OR = 1.001, 95% CI, 1.000-1.002, *P* = 8.01E-05, FDR = 0.03) exhibits a significant positive correlation with the risk of prostate cancer. When adjusting the false discovery rate (FDR) to 0.2, we identified associations between six immune cell phenotypes and the risk of prostate cancer. Among them, FSC-A on B cells (OR = 1.002, 95% CI, 1.001-1.002, *P* = 7.77E-04, FDR = 0.133), HLA DR on plasmacytoid dendritic cells (OR = 1.001, 95% CI, 1.000-1.001, *P* = 0.001, FDR = 0.133), CD14+ CD16- monocyte % monocytes (OR = 1.002, 95% CI, 1.001-1.003, *P* = 0.001, FDR = 0.133), and HVEM on effector memory CD4+ T cells (OR = 1.001, 95% CI, 1.000-1.002, *P* = 0.002, FDR = 0.169) are positively correlated with the risk of prostate cancer, while CD25 on IgD+ B cells (OR = 0.998, 95% CI, 0.997-0.999, *P* = 0.002, FDR = 0.169) and Monocytic Myeloid-Derived Suppressor Cells AC (OR = 0.999, 95% CI, 0.999-1.000, *P* = 0.002, FDR = 0.17) are negatively correlated with the risk of prostate cancer. Subsequently, a horizontal pleiotropy test was conducted using MR-Egger and MR-Presso in combination. No horizontal pleiotropy was detected in the above results, and Cochran’s Q test revealed no heterogeneity in all outcomes. Following that, scatter plots and funnel plots also supported these findings (**Supplementary Figures 1**, **2**). We further employed a heatmap for visual analysis of the research results. Initially, we filtered out the IDs of all positive results of immune cell phenotypes based on the p-values from the IVW method. Subsequently, different colors in **Figure 3** represent the p-values of sensitivity analysis results for each immune cell phenotype.

### The causal effect of I prostate cancer on immunophenotypes

In the reverse MR analysis, we identified some positive results, but after FDR correction (FDR < 0.05), no statistically significant results were observed. Similarly, after adjusting to FDR < 0.2, no meaningful results were detected.

## Discussion

Through a two-sample bidirectional MR study, we identified two immune cell phenotypes significantly associated with the risk of prostate cancer (FDR < 0.05). After adjusting for FDR < 0.20, an additional six immune cell phenotypes were found to be related to the risk of prostate cancer. In the reverse MR analysis, we also observed some positive results; however, after FDR correction, no significant correlations were identified.

Our study revealed a significant negative correlation between CD25 on naive-mature B cells and the risk of prostate cancer. Additionally, CD25 on IgD+ B cells also showed a negative correlation with the risk of prostate cancer, while FSC-A on B cells exhibited a positive correlation with the risk of prostate cancer. CD25 constitutes a component of the interleukin-2 (IL-2) receptor and is exhibited on the surface of diverse immune and non-immune cellular entities (25). The role of CD25 may vary significantly depending on its expression on different cell types. Regulatory T cells (Tregs) promote tumor progression, and CD25 is widely expressed on Tregs. Studies have found that depleting Tregs through anti-CD25 antibodies can exert an anti-tumor immune effect (26–28). However, recent research has found that agonists preserving the activity of CD25 can activate tumor-specific CD8 T cells, exerting an anti-tumor immune effect (29). These studies highlight the complex and crucial role of CD25 in tumor immunotherapy. Our study found that CD25 on naive-mature B cells and CD25 on IgD+ B cells are protective factors against prostate cancer. This finding aligns with similar conclusions from current research. Naive-mature B cells refer to B cells that have matured but have not been activated. In tumor immunity, naive-mature B cells may play an anti-tumor role by stimulating immune responses and assisting other immune cells (29). IgD+ B cells are a subset of B cells in the immune system, and they engage in immune responses through the surface expression of IgD. On B cells, IgD can coexist with other immunoglobulins, collectively regulating immune reactions (30). Studies have found the expansion of clonal B cells in both the blood and sentinel lymph nodes of prostate cancer patients, with a predominant presence of immature B cells in the blood (12). This reflects the protective role of B cells, including immature B cells, in prostate cancer.

Our study identified HLA DR on CD14- CD16-, CD14+ CD16- monocyte %monocyte, and HLA DR on plasmacytoid Dendritic Cell as risk factors for the incidence of prostate cancer. CD14 and CD16 are surface markers on immune cells, playing crucial roles in signal recognition, signal transduction, and enhancement of immune responses. They exert significant functions in both tumor and non-tumor diseases (31, 32). In the Monocyte panel, they were identified based on HLA-DR positivity. Pavlovic et al. has found reduced expression of the monocyte HLA-DR molecule in prostate cancer (33). A previous study indicated that enhancing monocyte function in the human body can be achieved by upregulating HLA-DR, contributing to anti-prostate cancer effects (34). Monocytes were categorized into classical cells (CD14+CD16−), non-classical cells (CD14−CD16+), and intermediate cells (CD14+CD16+) (21). Although the role of HLA-DR on CD14- CD16- is rarely mentioned, a recent study (35) still highlights its significant involvement in schizophrenia, warranting further attention. Currently, there is no dedicated study on the specific mechanism of HLA DR on CD14- CD16- in the development of prostate cancer. Further research is needed to validate our findings and explore the potential mechanisms involved. Plasmacytoid Dendritic Cells are multifunctional immune cells, and their clinical significance in the tumor microenvironment (TME) remains unclear (36), previous studies have found the immunosuppressive role of plasmacytoid dendritic cells in gastric cancer (37). The mechanisms by which they function in prostate cancer still require further exploration. Our study also found that Monocytic Myeloid-Derived Suppressor Cells (M-MDSCs) may be a potential protective factor in the development of prostate cancer. M-MDSC and polymorphonuclear MDSC (PMN-MDSC) are two main cellular subtypes of myeloid-derived suppressor cells (MDSCs). Morphologically, M-MDSCs resemble monocytes, while PMN-MDSCs have a multi-lobed nucleus similar to polymorphonuclear (PMN) cells. Moreover, these two subtypes express different surface molecules, and their distribution varies across different tumors (38). Previous studies have found that PMN-MDSCs contribute to the progression and immune evasion of prostate cancer (39, 40). Idorn et al. have identified increased expression of M-MDSCs in patients with castration-resistant prostate cancer, suggesting its involvement in the immune suppressive environment of prostate cancer patients (41). Further specific research is needed to elucidate these findings.

The strengths of our study include the first-time application of MR methods to investigate the relationship between immune cell phenotypes and prostate cancer. Our conclusions were derived under strict examination of horizontal pleiotropy, reducing the interference of confounding factors and the impact of reverse causality on the results. Additionally, our study identified immune cell phenotypes significantly associated with prostate cancer, which have been less explored in previous research. This may provide new insights for exploring potential immunotherapeutic targets in prostate cancer. Our study also has some limitations. Although we included 731 immune cell phenotypes in our research, there are still some immune cell phenotypes that could not be analyzed due to data limitations. Additionally, since the data sources are predominantly of European descent, limited to adults, and do not support stratification by gender and age, this may impact the generalizability and accuracy of the results. This study is based on a cohort study of individuals from Sardinia. The Sardinian population possesses unique genetic characteristics, and the study conclusions may not be applicable to broader populations. Future validation in larger and more diverse patient cohorts is necessary to ensure the robustness and generalizability of our conclusions. Furthermore, the selection of instrumental variables for immune cell phenotypes (*P* < 1×10−5) and the interpretation of reverse results (FDR < 0.2) are not as stringent. Finally, we hope that future research will involve larger sample sizes and more comprehensive Mendelian randomization studies to further explore the relationship between immune cell phenotypes and prostate cancer.

## Conclusions

This study has revealed causal relationships between immune cell phenotypes and prostate cancer, supplying novel insights that might aid in comprehending the pathogenic mechanisms of prostate cancer and identifying potential therapeutic targets.

## Data availability statement

The original contributions presented in the study are included in the article/**Supplementary Material**. Further inquiries can be directed to the corresponding author.

## Ethics statement

Ethical approval was not required for the study involving humans in accordance with the local legislation and institutional requirements. Written informed consent to participate in this study was not required from the participants or the participants’ legal guardians/next of kin in accordance with the national legislation and the institutional requirements.

## Author contributions

XH: Writing – review & editing, Methodology, Software, Writing – original draft. CR: Writing – original draft, Software. HZho: Writing – original draft, Investigation. ML: Data curation, Writing – review & editing. HZha: Data curation, Writing – review & editing. XL: Project administration, Supervision, Writing – review & editing.
