# Supplementary material for: Association between circulating immune cells and the risk of prostate cancer: a Mendelian randomization study
Source: Front Endocrinol (Lausanne). 2024 Feb 9;15:1358416. doi: 10.3389/fendo.2024.1358416 (PMC10884280; doi:10.3389/fendo.2024.1358416)
Supplement: Supplementary file 1 [file Image_1.pdf]

Supplementary Material for

Association between circulating immune cells and the the risk of prostate cancer

1. Supplementary Figures

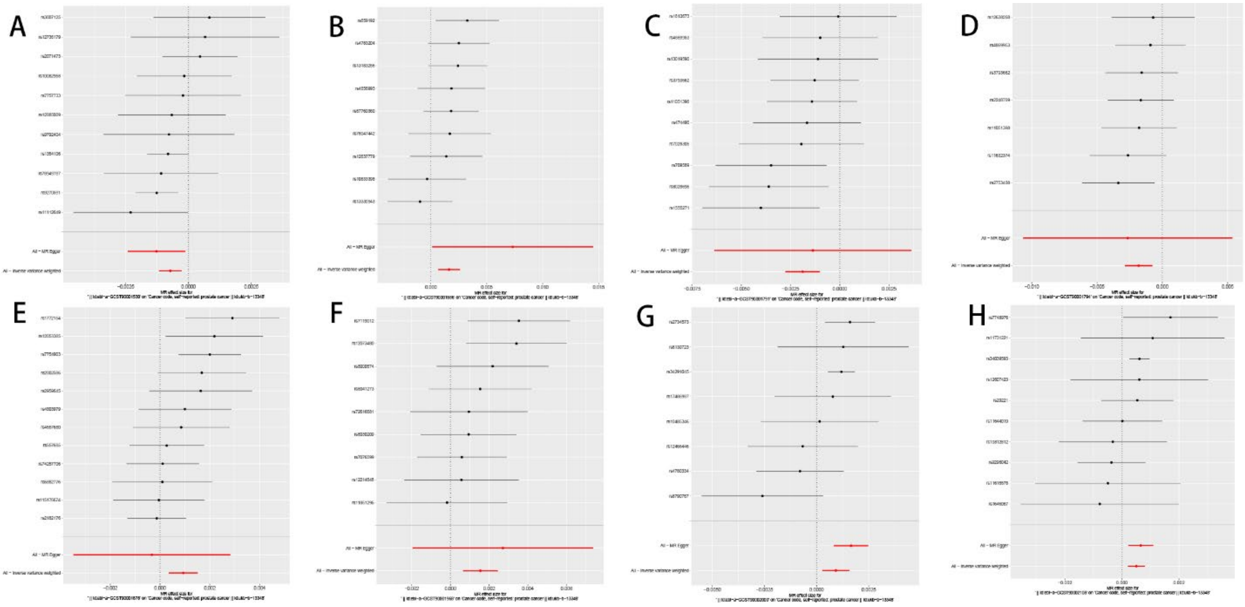

Supplementary Figure S1. funnel plots **(A)** Monocytic Myeloid-Derived Suppressor Cells AC on prostate cancer; **(B)** CD14+ CD16- monocyte %monocyte on prostate cancer; **(C)** CD25 on naive-mature B cell on prostate cancer; **(D)** CD25 on IgD+ B cell on prostate cancer; **(E)** HVEM on Effector Memory CD4+ T cell on prostate cancer; **(F)** FSC-A on B cellon prostate cancer; **(G)** HLA DR on CD14- CD16- on prostate cancer; **(H)** HLA DR on plasmacytoid Dendritic Cell on prostate cancer;

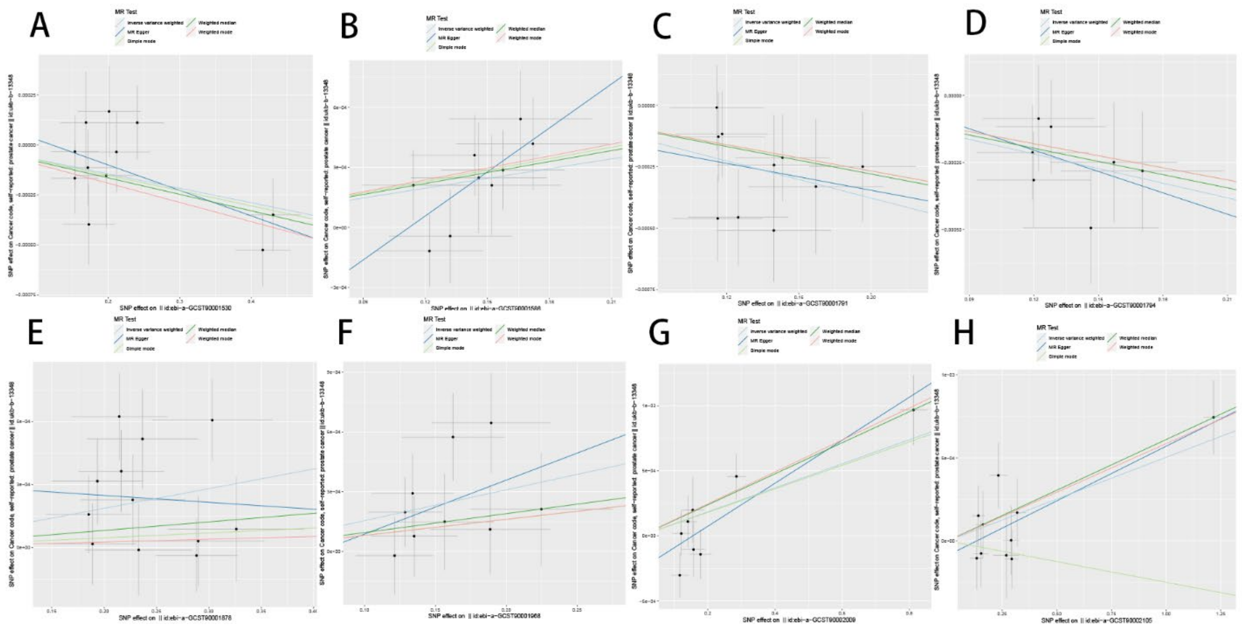

Supplementary Figure S2. scatter plots **(A)** Monocytic Myeloid-Derived Suppressor Cells AC on prostate cancer; **(B)** CD14+ CD16- monocyte %monocyte on prostate cancer; **(C)** CD25 on naive-mature B cell on prostate cancer; **(D)** CD25 on IgD+ B cell on prostate cancer; **(E)** HVEM on Effector Memory CD4+ T cell on prostate cancer; **(F)** FSC-A on B cellon prostate cancer; **(G)** HLA DR on CD14- CD16- on prostate cancer; **(H)** HLA DR on plasmacytoid Dendritic Cell on prostate cancer;
